# The potential of a constellation of low earth orbit satellite imagers to monitor worldwide fossil fuel CO_2_ emissions from large cities and point sources

**DOI:** 10.1186/s13021-020-00153-4

**Published:** 2020-09-04

**Authors:** Franck Lespinas, Yilong Wang, Grégoire Broquet, François-Marie Bréon, Michael Buchwitz, Maximilian Reuter, Yasjka Meijer, Armin Loescher, Greet Janssens-Maenhout, Bo Zheng, Philippe Ciais

**Affiliations:** 1 Laboratoire des Sciences du Climat et de L’Environnement, CEA-CNRS, UVSQ-Université Paris Saclay, Gif-sur-Yvette, France; 2 Canadian Centre for Meteorological and Environmental Prediction, 2121 Transcanada Highway, Dorval, QC H9P 1J3 Canada; 3 Key Laboratory of Land Surface Pattern and Simulation, Institute of Geographical Sciences and Natural Resources Research, Chinese Academy of Sciences, Beijing, China; 4 Institute of Environmental Physics (IUP), University of Bremen FB1, Otto Hahn Allee 1, 28334 Bremen, Germany; 5 European Space Agency (ESA), Noordwijk, Netherlands; 6 Joint Research Centre, Directorate Sustainable Resources, European Commission, Transport & Climate, Via Fermi 2749, 21027 Ispra, Italy

**Keywords:** Satellite imager, PMIF global inversion system, Anthropogenic CO_2_ emissions, Posterior uncertainty

## Abstract

**Background:**

Satellite imagery will offer unparalleled global spatial coverage at high-resolution for long term cost-effective monitoring of CO_2_ concentration plumes generated by emission hotspots. CO_2_ emissions can then be estimated from the magnitude of these plumes. In this paper, we assimilate pseudo-observations in a global atmospheric inversion system to assess the performance of a constellation of one to four sun-synchronous low-Earth orbit (LEO) imagers to monitor anthropogenic CO_2_ emissions. The constellation of imagers follows the specifications from the European Spatial Agency (ESA) for the Copernicus Anthropogenic Carbon Dioxide Monitoring (CO2M) concept for a future operational mission dedicated to the monitoring of anthropogenic CO_2_ emissions. This study assesses the uncertainties in the inversion estimates of emissions (“posterior uncertainties”).

**Results:**

The posterior uncertainties of emissions for individual cities and power plants are estimated for the 3 h before satellite overpasses, and extrapolated at annual scale assuming temporal auto-correlations in the uncertainties in the emission products that are used as a prior knowledge on the emissions by the Bayesian framework of the inversion. The results indicate that (i) the number of satellites has a proportional impact on the number of 3 h time windows for which emissions are constrained to better than 20%, but it has a small impact on the posterior uncertainties in annual emissions; (ii) having one satellite with wide swath would provide full images of the XCO_2_ plumes, and is more beneficial than having two satellites with half the width of reference swath; and (iii) an increase in the precision of XCO_2_ retrievals from 0.7 ppm to 0.35 ppm has a marginal impact on the emission monitoring performance.

**Conclusions:**

For all constellation configurations, only the cities and power plants with an annual emission higher than 0.5 MtC per year can have at least one 8:30–11:30 time window during one year when the emissions can be constrained to better than 20%. The potential of satellite imagers to constrain annual emissions not only depend on the design of the imagers, but also strongly depend on the temporal error structure in the prior uncertainties, which is needed to be objectively assessed in the bottom-up emission maps.

## Background

Cities, thermal power plants and industrial sites are the largest emitters of fossil fuel CO_2_ that causes global warming [10]. Monitoring CO_2_ emissions from these hotspots is therefore a priority for assessing the effectiveness of greenhouse gas (GHG) emissions reduction policies. Knowledge of the magnitude and spatial and temporal variability of CO_2_ emissions at the regional scale is also critical in unraveling the natural sources and sinks of the carbon cycle. Existing inventories of CO_2_ emissions from fossil fuel combustion can provide accurate information at the national and annual scales in the most developed countries, but they are subject to many uncertainties in developing countries and at the scale of cities, individual point sources (e.g. power plants) and regions due to a lack of local statistical data on energy and fuel consumption [1, 13]. Furthermore, regulations and commitments are taken at the scale of cities and individual sectors. It is thus necessary to develop systems capable of providing frequent and accurate estimates of CO_2_ emissions at the scale of anthropogenic emission hotspots.

The method for monitoring anthropogenic CO_2_ emissions from these hotspots, called atmospheric inversion, combines prior information on local CO_2_ emissions from inventories with observations of atmospheric CO_2_ concentrations sensitive to emissions, using atmospheric transport models and statistical inversion (e.g.[5, 17]). The underlying idea is that CO_2_ concentration measurements can be used to characterize downwind CO_2_ plumes from large emission hotspots. Dense observations of the CO_2_ mole fractions in the atmosphere facilitate the characterization of CO_2_ emission plumes that is used to retrieve emission estimates. In situ measurements of CO_2_ mole fractions from surface networks, aircraft campaigns and mobile platforms have been used to quantify the emissions from cities and power plants [5, 17, 30–32]. However, such networks are deployed for few cities and point sources only. The cost for maintaining a given urban network of to conduct regular campaigns around a given source limits for their deployment. Furthermore, the atmospheric inversions can be hampered by the discrete and limited spatial (both horizontally and vertically) sampling of fixed networks [30] or by the lack of temporal representativeness of the infrequent mobile campaigns. Alternatively, vertically integrated columns of dry-air mole fractions of CO_2_ (XCO_2_) from satellites offer the advantage of providing full spatial coverage of the plumes of individual sources and covering a wide range of sources over the world, in cloudless conditions. For example, Nassar et al. [22] used the XCO_2_ observations from OCO-2 to quantify CO_2_ emissions from seven middle- to large-sized coal power plants from USA, India and South Africa. Wu et al. [37] estimated CO_2_ emissions from 20 cities across several continents using the XCO_2_ observations from OCO-2. Zheng et al. [38] developed an algorithm for the automatic detection of plumes and inversion of fossil fuel CO_2_ emissions in the OCO-2 XCO_2_ data applied it to estimate the emissions from 46 cities and power plants in China using 5 years of OCO-2 data. These studies and some others (e.g. [14, 29]) reveal that a limited amount of clear transects of XCO_2_ plumes from cities or plants are currently detected in OCO-2 observations so that the real data from current on-orbit satellites keep on being hardly used to estimate anthropogenic CO_2_ emissions.

. In this context, there is growing interest in developing satellite imager instruments capable of providing high spatial resolution measurements of vertically integrated columns of dry air mole fractions of CO_2_ (XCO_2_) over the globe (e.g.[4, 8]). In particular, the European Spatial Agency (ESA) is studying the potential of the Copernicus Anthropogenic Carbon Dioxide Monitoring (CO2M) mission consisting of a constellation of CO_2_ imagers to monitor anthropogenic CO_2_ emissions.

Many studies were conducted to assess the potential of satellite imagery to reduce uncertainties in fossil CO_2_ emissions at the regional (e.g. [23]). and city (e.g. [6]). scales, and for point sources (e.g.[4, 15, 27, 34]). These studies showed that satellite images must have a high spatial resolution between 1–20 km^2^ to accurately monitor CO_2_ plumes from cities and point sources with sufficient cloudless observations (e.g.[4]). They also indicated that a high retrieval precision (less than 1 ppm) of XCO_2_ is required to quantify the enhancement of the CO_2_ column in the plumes relative to the background even for large cities like Paris and Berlin [6, 25]. Among the important factors associated with the mission specifications, the swath width of the satellites was also shown to have to be larger than 100 km to frequently sample plumes of anthropogenic CO_2_ emissions with only a few satellites. Based on these preliminary results, CO2M satellites were considered to follow a late morning orbit (Equator-crossing time at ~ 11:30, local time descending node) when there are, on average, fewer clouds, lower wind speeds, and higher anthropogenic CO_2_ emissions than in early afternoon [12]. The most likely option for the width of the CO2M instrument is 300 km, but a range of options from 150 to 400 km is also being discussed.

However, most of the existing studies have focused on specific, and generally very large cities and plants and do not provide a global picture of the ability of satellite imagery to constrain CO_2_ emissions across a full range of emission hotspots (with various emission rates and areas, and distances to other emission hotspots). In this context, Wang et al. [35] presented the “Plume-Monitoring Inversion Framework” (PMIF) global inversion system to assess the potential of satellite imagery to monitor CO_2_ emissions from all emission hotspots (including cities and power plants, which are called “clumps” following the global classification of such hotspots based on a high-resolution emission map by [36] around the globe and during a full year. The definition of 11,314 clumps from the CO_2_ emission map Open Source Data Inventory of Anthropogenic CO_2_ (ODIAC) with a 1-km × 1-km spatial resolution was described in Wang et al. [36]. They represent all the CO_2_ sources that can generate an XCO_2_ enhancement of at least 0.5 ppm under optimistic meteorological conditions without wind. In ODIAC, the sum of fossil fuel CO_2_ emissions from all clumps of the globe account for about 72% of the global total. The PMIF inversion system is based on a Bayesian statistical framework, a global high-resolution emission map, a global dataset on wind fields, a realistic sampling of satellite data, as well as errors on individual XCO_2_ retrievals. The Bayesian framework corrects a prior estimate of the control variables (here emission budgets for individual cities or power plants) from inventories, based on the atmospheric data, to derive a posterior estimate. The system assesses the uncertainties in the posterior estimate ("the posterior uncertainties”), which is a function of the uncertainties in the prior estimate (“the prior uncertainties”), of the observation sampling and errors and of the atmospheric transport. The design of the PMIF inversion system relies on a complex combination of short and regional assimilation windows and on some simplifications compared to traditional inversion systems for a given assimilation window. This is necessary to limit the computational costs because of the large number of assimilated observations and the large number of control variables when covering the globe and a full year. Among the most critical simplifications of PMIF is the choice of the transport model: the CO_2_ plumes emitted by the CO_2_ sources are modeled using a simple Gaussian plume model that only takes into account the local mean wind field and the mean emissions, both over the three hours (i.e. 8:30–11:30 for CO2M mission) before the satellite overpass (~ 11:30LT). The Gaussian plume model can often hardly fit with actual plumes over long distances (due to variations in the wind field, topography, vertical mixing etc.) but is shown, when driven with suitable parameters, to provide a satisfactory simulation of the plume extent and amplitudes, which appear to be the main drivers of the targeted computations of uncertainties in the emissions in our OSSE framework [35]. The choice of focusing on the three hours before the satellite overpasses is driven by the analysis of Broquet et al. [6] indicating that the temporal representativeness of the detectable part of the plumes from a large city like Paris is about few hours when using a satellite imaging concept similar to CO2M. In addition, PMIF ignores uncertainties in the natural fluxes and in diffuse anthropogenic emissions outside the clumps.

Wang et al. [35] used PMIF to assess the performance of a single satellite imager for monitoring CO_2_ emissions for the full range of clumps and meteorological conditions representative of the whole globe and a full year. They found that only the clumps with annual emission budgets higher than 2 MtC per year can potentially have their emissions between 8:30 and 11:30 constrained with a posterior uncertainty less than 20% for more than 10 overpasses within the year (ignoring the temporal correlations in the prior uncertainty). They also aggregated the posterior uncertainties in time to investigate the potential of satellite observations to constrain daily and annual emissions. Their results revealed that the hypotheses on the temporal correlations of prior uncertainties have a critical impact on the potential of one satellite imager to constrain daily and annual emissions for individual clumps. Typically, annual budgets for cities with an annual budget larger than 10MtC yr^−1^ can be constrained to less than 15% [35] with a strong temporal auto-correlation (prior uncertainties in hourly emissions being fully correlated within the day and the auto-correlation between the same hours from different days being described by an exponential decaying function with 20-day correlation length), against 25% with a moderate temporal auto-correlation (with a 7-day correlation length for day-to-day auto-correlation and a 12-h correlation length for hour-to-hour auto-correlations). Here, we extend the analysis of the inversion performances to cover the most likely scenarios for the CO2M mission, with a constellation of one to four LEO imagers. The other goal of the paper is to investigate the impacts of the major parameters of the instrument in CO2M mission, in order to support the optimization of its design: the width of the swath and the precision of a single XCO_2_ retrieval. The metrics used are the posterior uncertainty in the 3 h (8:30–11:30) mean emissions and in the annual budget. This assessment is detailed for groups of clumps gathered as a function of their annual emission budgets and across all regions of the world.

The manuscript is organized as follows: Sect. 2 briefly reminds the principles, data and practical implementation of the inversion system developed by Wang et al. [35]. Section 3 presents an assessment of the performance of inversions with the assimilation of satellite data from one to four imagers and different options regarding the instrument swath and precision of XCO_2_ retrievals. Section 4 discusses the factors that may explain the variability of the results found on the inversions and some limitations of the inversion system.

## Methods

The PMIF follows the traditional Bayesian statistical framework of the atmospheric inversions, updating a prior statistical estimates (x^b^) of a set of control variables **x** that corresponds to emission budgets for the clumps. The update relies on the observations **y**^o^, on an observation operator ***x*** → ***y*** = **Mx** + ***y***^fixed^ that links the control space and the observation space, and on the statistics of the so-called “observation error”. Here, ***y***^fixed^ is the signature in the observation space of the CO_2_ fluxes that are not controlled by the inversion. Since we ignore uncertainties in these fluxes, this term has no impact in the inversion computations and is ignored hereafter (and we call **M** the observation operator in the following). The observation error is a combination of all sources of errors in the observation operator and in the observations themselves, i.e. all source of misfits between the modeled and observed concentration other than errors in the prior estimate of the control variables. The prior uncertainties and the observation errors are assumed to be unbiased and to follow the Gaussian distributions *N*(***0***, **B**) and *N*(***0***, **R**), where **B** and **R** represent the error covariance matrices for the prior uncertainties and observation errors. The statistical estimate of the control variable updated in the Bayesian framework, called the posterior estimate, follows a Gaussian distribution *N*(***x***^a^, **A**), with ***x***^a^ being the mean and **A** being the posterior uncertainty covariance matrix. The solution of the inversion is derived by:

1 $$ {\mathbf{A }} = \, \left( {{\mathbf{ B}}^{{ - {1}}} + {\mathbf{ M}}^{{\mathbf{T}}} {\mathbf{R}}^{{ - {1}}} {\mathbf{M }}} \right)^{{ - {1}}} $$

2 $$ \varvec{x}^{{\text{a}}} = \varvec{x}^{{\text{b}}} + {\mathbf{AM}}^{{\text{T}}} {\mathbf{R}}^{{ - {1}}} \left( {\varvec{y}^{{\text{o}}} {-}{\mathbf{M}}\varvec{x}^{{\text{b}}} {-}\varvec{y}^{{{\text{fixed}}}} } \right) $$

The PMIF system is described in detail in Wang et al. [35]. Here we only summarize the main elements.

PMIF solves for a scaling factor for the 3-h mean emissions between 8:30 and 11:30 and a scaling factor for the emissions during the rest time of the day (0:00–8:30 plus 11:30–24:00) for each day over 1 year and for all the clumps over the globe. The observation operator is null for the emissions between 0:00–8:30 plus 11:30–24:00 since we assume these emissions does not raise any significant XCO_2_ signal in the CO2M satellite images at 11:30. The part of the observation operator **M** corresponding to emissions between 8:30 and 11:30 has two components. The first one (**M**_inventory_) describes the spatial distribution of emissions within the area of a clump and the temporal variation of the emissions within the time window: ***x*** → ***E*** = **M**_inventory_***x***. In PMIF, the spatial distribution of the emissions are based on the Open Source Data Inventory of Anthropogenic CO_2_ Emission [24] for the year 2016, and the temporal variation of the emissions are derived from the monthly profile from ODIAC and the weekly and diurnal (at hourly resolution) profiles from the Temporal Improvements for Modeling Emissions by Scaling (TIMES) product [20]. The second component of **M** (**M**_plume_) simulates the plumes of XCO_2_ enhancement above the background downwind given clumps: ***E*** → ***y*** = **M**_plume_***E***. **M**_plume_ is the aggregation of the XCO_2_ enhancement generated by each emitting pixel of the ODIAC map within a given clump. For each emitting pixel, we assume that the plume of XCO_2_ enhancement downwind has a Gaussian shape:

3 $${\varvec{y}}\left(i,j\right)=\alpha \frac{E}{\sqrt{2\pi }{\sigma }_{j}u}{e}^{-\frac{{j}^{2}}{2{\sigma }_{j}^{2}}}$$

where ***y*** is the XCO_2_ enhancement (in ppm) at a downwind location (*i*, *j*). The *i*-direction is parallel to the local mean wind direction and the *j*-direction is perpendicular to that direction. In this study, the wind field is taken from the Cross-Calibrated Multi-Platform (CCMP) gridded surface wind product for the year 2008 [3]. The CCMP product consists in 6-hourly gridded wind vectors at a horizontal resolution of 0.25 degree. It is based on the combination of Version-7 RSS radiometer, QuikSCAT and ASCAT scatterometer and moored buoy wind data with ERA-Interim model wind fields. σ_j_ is a function of downwind distance *i*, similar to that used by Ars et al. [2]. α is a coefficient that converts the computed CO_2_ enhancement (in g/m^2^) in the XCO_2_ unit of ppm, assuming a standard surface pressure of 1013 hPa and a standard molar mass of dry air of 28.97 g mol^−1^.

In PMIF, we use the AMS (Annual component and Moderately correlated Sub-annual component) configuration of the prior uncertainty in the emission budgets, as described in Wang et al. [35], because it has a plausible configuration on the temporal auto-correlation in prior uncertainties according to the comparison between inventories and actual emission proxies [16, 35]. In practice, it assumes that the prior uncertainty in the emissions for each clump has two components. The first one is an annual component that is fully auto-correlated in time over 1 year (i.e. a bias in time), whose amplitude follows an unbiased Gaussian distribution ~ *N*(0, 29%). This component of the prior uncertainty accounts for unknown information about the city or point source that does not change over time. The second one is a sub-annual uncertainty component bearing some temporal auto-correlations. The temporal auto-correlation between this component of the uncertainties in hourly emissions at two instants distant by Δd days and Δh hours is formulated as r = exp(-Δh/τ_1_) × exp(-Δd/τ_2_), where τ_1_ = 12 h and τ_2_ = 7d. This component of the prior uncertainty follows the distribution ~ *N*(0, 49%) for the 3 h emissions between 8:30 and 11:30 and ~ *N*(0, 38%) for the rest 21 h emissions of the day. This second component (variable) is for emission variations that are not accounted for in the prior information of emissions, such as those linked to weather systems (heating) or specific variable activities. The total uncertainty of emissions for each control variable is the square root of the quadratic sum of the two uncertainty components.

**Fig. 1:**
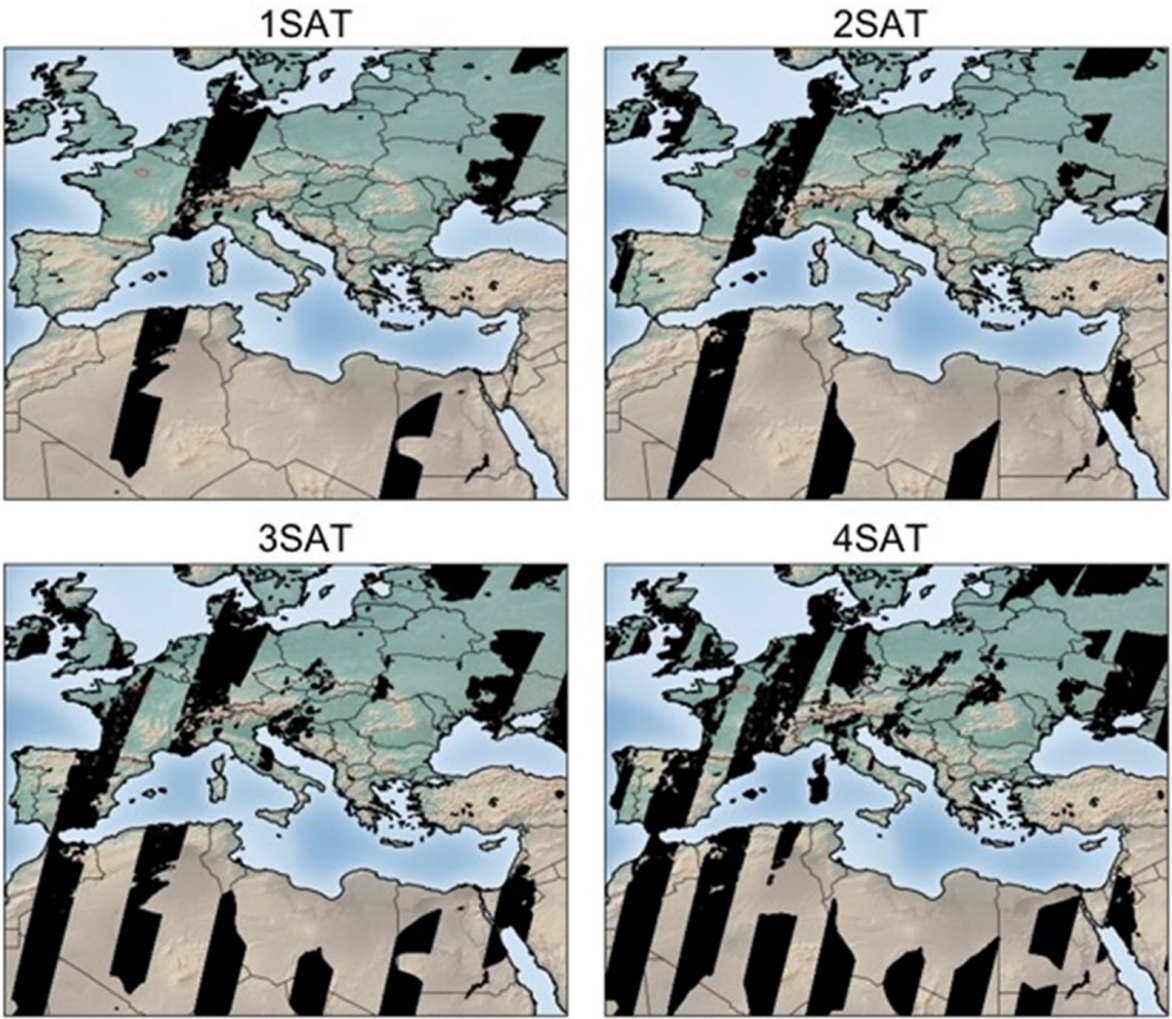
Representation of the spatial coverage of 1 to 4 satellite constellations for a 24-h period on 12 April 2008. The black pixels show the location of valid (free of heavy cloud and aerosols) satellite observations

PMIF assimilates satellite pseudo-observations corresponding to the simulation of a one-year global sampling (using data corresponding to the year 2008) by sun-synchronous LEO satellites of the CO2M mission. The orbit has a repeat cycle of 16 days with an Equator-crossing time of 11:30. As Eq. (1) shows that the posterior uncertainty only depends on prior and observation error covariance matrices, on the observation operator, and implicitly on the structure of the observation vector (i.e., on the time, location and representation of the observations through **M**), we only consider the precision and sampling (time, location and spatial resolution) of the synthetic satellite observations. The precision of individual XCO_2_ retrievals from the crude radiance measurements (called the random measurement error) is simulated following the same formulation as in Buchwitz et al. [7] to simulate the impact of changes in the surface and atmospheric conditions, but with updated parameters to model the impact of SNR and other instrumental specifications for CO2M missions. For the reference CO2M configuration, the random measurement error is 0.7 ppm for vegetation albedo and solar zenith angle (SZA) 50º. The width of the swath in the reference configuration is 300 km and the horizontal resolution of the CO2M instruments is 2 km × 2 km. Various options for the random measurement error and swath width are also being considered (detailed below). The Moderate Resolution Imaging Spectroradiometer (MODIS) Terra MOD35 cloud and aerosol data product (https://modis-atmos.gsfc.nasa.gov/MOD35_L2/) was used to simulate cloud/aerosol-contaminated XCO_2_ retrievals (see [7] for more details). Only “good” XCO_2_ data that are cloud-free and for which the sum of the retrieved aerosol optical depth (AOD) at NIR wavelength and atmosphere cirrus optical depth (COD) is less than 0.3, are used in the inversions. These data are referred to as “clear sky” data hereafter. The presence of clouds and aerosol induce data gaps in the simulated XCO_2_ fields (Fig. 1).

In this study, we simulate the sampling of XCO_2_ observations from constellations consisting of one to four CO2M imagers. The spatial coverage of the XCO_2_ observations from the constellations are shown in Fig. 1. The simulations for these constellations are based on combinations of the simulations for one to four satellites on the same helio-synchronous orbit, satellites being equally spaced in the orbit for a given constellation (Additional file 1: Fig. S1). The constellation of 3 satellites includes the satellite that is used to test the 1-satellite configuration of CO2M. Similarly, the two imagers in 2-satellite case are included in the 4-satellite constellation (Additional file 1: Fig. S1). But the 3-satellite constellation and the 4-satellite constellation do not have any satellite in common.

We conduct two sets of observing system simulation experiments (OSSEs) to investigate the potential of satellite observations in constraining the emissions at various time scales. The potential of satellite imagery is assessed in terms of the posterior uncertainty in the emission budgets for each clump. Firstly, we conduct an OSSE for each 8:30–11:30 window of the year seperately (called INV-3 h hereafter), ignoring the potential to cross or extrapolate the information between days based on the temporal auto-correlation in the prior uncertainty. In practice, this is done by ignoring the temporal covariances (off-diagonal entries) in the **B** matrix. The 3 h mean emissions are considered as *significantly* constrained when the posterior uncertainty is less than 20%. Physically, it means there are at least one satellite overpass in the vicinity of the clumps and a sufficient number of XCO_2_ observations with adequate precision within the XCO_2_ plumes generated from the clumps. The number of 8:30–11:30 windows for which the mean emissions are significantly constrained is denoted as N20 for each clump. Then we conduct a second set of OSSEs, in which the system fully exploits the temporal auto-correlations in **B** to cross information from different overpasses, and extrapolate it to constrain emissions whose XCO_2_ signature is not observed (that for the other 21 h within a day and the days with no satellite observations near the clump). We analyze the posterior uncertainty for the 3 h (8:30–11:30) mean emissions on all days over one year, and also for annual budget by aggregating the posterior uncertainty covariance matrix **A** in the second set of OSSEs. This set of OSSEs are referred to as INV-annual.

In addition to the number of satellites, the swath width and random errors of XCO_2_ observations may impact the inversion performance while some options are still under discussion for CO2M. While the reference computations with the 1 to 4 satellite constellation are led with swath with 300 km width, and the default simulation of the random measurement error for CO2M, we change these parameters in the configuration of the imagers and quantify their impacts. First, we use the first and third satellites from the 4-satellite constellation and reduce the swath width of these satellites to 150 km. The results are compared with those obtained with one satellite and a swath width of 300 km. Then, we reduce the random measurement error in the 3-satellite constellation (with a default swath width of 300 km) by 20% or 50%, respectively, to investigate the benefit of improving the measurement precision in constraining the emissions. We also increase the random measurement error by 43% and 71%, such that the resulting random measurement errors are comparable to the typical precision of XCO_2_ achieved by OCO-2 or simulated for GeoCARB [11],https://cdn.eventsforce.net/files/ef-xnn67yq56ylu/website/9/739_berrien_moore_-_geostationary_carbon_cycle_observatory__geocarb_-unraveling_the_carbon-weather-climate_system.pdf). Such a correction of the instrument precision is assumed to scale homogeneously the random measurement error for all the XCO_2_ data:

4 $${\varepsilon }_{i}={\varepsilon }_{o}\times (1-\mathrm{\alpha })$$

where ε_*o*_ is the random error in the reference simulation for a given observation (typically 0.7 ppm for vegetation albedo and SZA 50º), α equals to 20%, 50%, -43% and -71% in corresponding scenarios. ɛ_*i*_ is the resulting random measurement error, equaling to 0.56, 0.35, 1.0 and 1.2 ppm for vegetation albedo and SZA 50º in the four scenarios respectively.

## Results

**Fig. 2:**
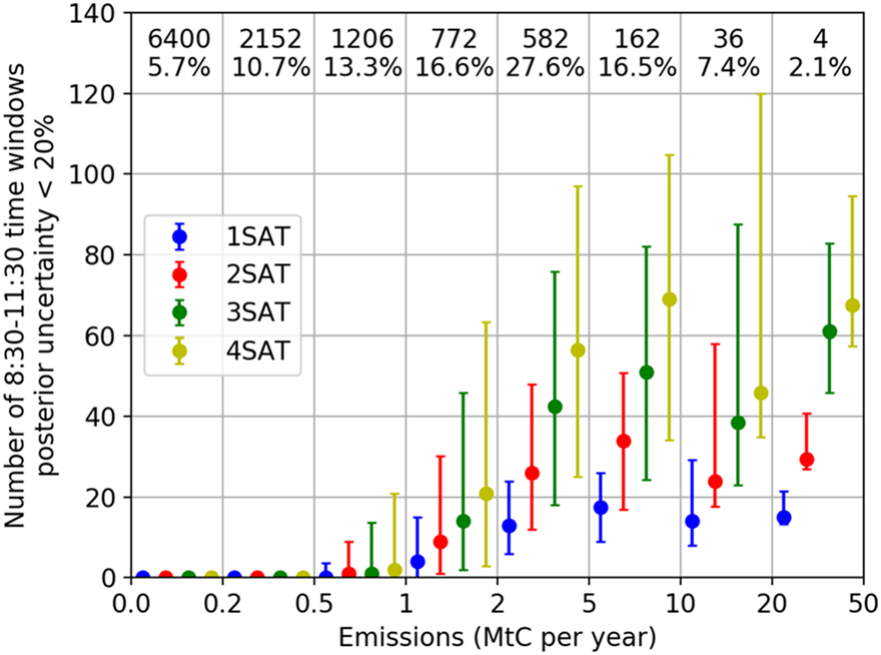
Number of 8:30–11:30 time windows in a year (here for the year 2008) for which the posterior uncertainty in the 3 h mean emissions are less than 20% (N20) in INV-3 h. The results are binned according to the clump annual emission with bin limits given on the x-axis of the figure. Numbers within the figure indicate the number of clumps and the fraction of total CO_2_ emissions generated from all the clumps within each bin. Dots and error bars are the median and interquartile range of N20

Figure 2 shows the median and interquartile range of N20 calculated for various clumps ranked by their annual emissions and for different satellite constellations in INV-3 h with reference configuration of the satellite imagers, i.e. the swath width being 300 km and the typical random measurement error being 0.7 ppm (for vegetation albedo and SZA 50º). It shows that only clumps whose annual emission budget are larger than 0.5 MtC per year have at least one 8:30–11:30 time window over the year for which the mean emissions can be constrained with the posterior uncertainty smaller than 20%. These clumps account respectively for 24.4% of the total number of clumps and for 83.6% of the fraction of total CO_2_ emissions covered by all clumps. N20 values tends to increase with the emission budget of clumps. The N20 median values are respectively 1, 14, 43, 51, 39 and 61 for the 0–1, 1–2, 2–5, 5–10, 10–20 and 20–50 MtC per year emission bins for the three-satellite constellation, due to the fact that the atmospheric plume generated by large emission clumps (in terms of emission budget) is easier to be filtered from the measurement noise than that by small clumps. The values of N20 tend to increase proportionally with the number of satellites for all emission bins. For example, the N20 median is 18, 34, 51 and 69 with 1, 2, 3 and 4 satellites for the clumps in the emission bin of 5–10 MtC per year. This increase is mostly linear for all emission bins except for those of 10–20 (e.g. Paris, France) and 20–50 (e.g. Beijing, China) MtC per year for which the increase in the N20 values is much larger between 2 and 3 satellites than between 3 and 4 satellites. This exception seems to be a statistical artefact linked to the small number of clumps in this category and to the fact that the simulated satellite overpasses are different for the various constellation (Fig. 1 and Additional file 1: Fig. S1).

**Fig. 3:**
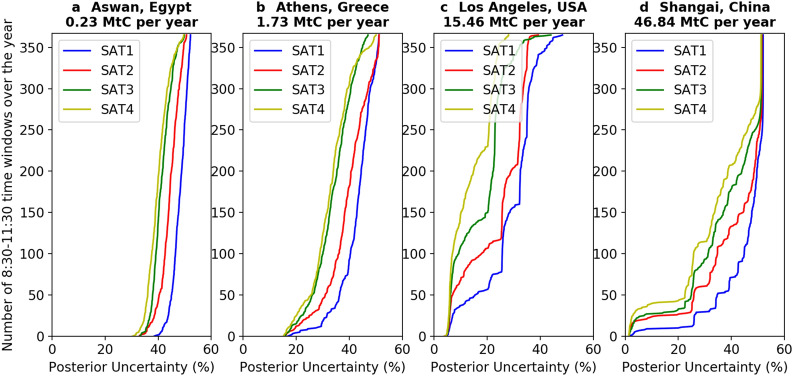
Cumulative distribution of posterior uncertainty for 3 h mean emissions over one year for four cities with different annual budget of CO2 emissions in INV-annual. The y-axis indicates the number of 8:30–11:30 time windows for which the posterior uncertainty of the 3 h mean emission is less than the value of the x-axis

The impact of the number of satellites on the posterior uncertainties in 3 h mean emissions in INV-annual can be seen in Fig. 3 that shows the cumulative distribution of the number of 8:30–11:30 time windows when the posterior uncertainty is less than a threshold varying between 0 and 60% for a few exemplary cities with different annual budgets of CO_2_ emission. Figure 3 shows that the cumulative number of 8:30–11:30 time windows in Los Angeles (USA; Fig. 3c) under a given value of posterior uncertainty is greater than in Shanghai (China; Fig. 3d), even though Shanghai has a much higher CO_2_ emission budget. More generally, at the regional level, the best results in terms of posterior uncertainty are obtained in North America where the median values of N20 are generally larger than those found in the other regions, while poorer results are found in Asia where the median values of N20 are less than 50 for all emission bins except for the 20–50 MtC per year emission bin (Additional file 1: Figure S2). Wang et al. [35] showed that the frequency of clear-sky days is an important driver of the N20 values and the posterior uncertainty in mean 3 h emissions. They also showed that the clumps in North America are located at places where there are generally more clear-sky days during the year than at the locations of the clumps in Asia.

**Fig. 4:**
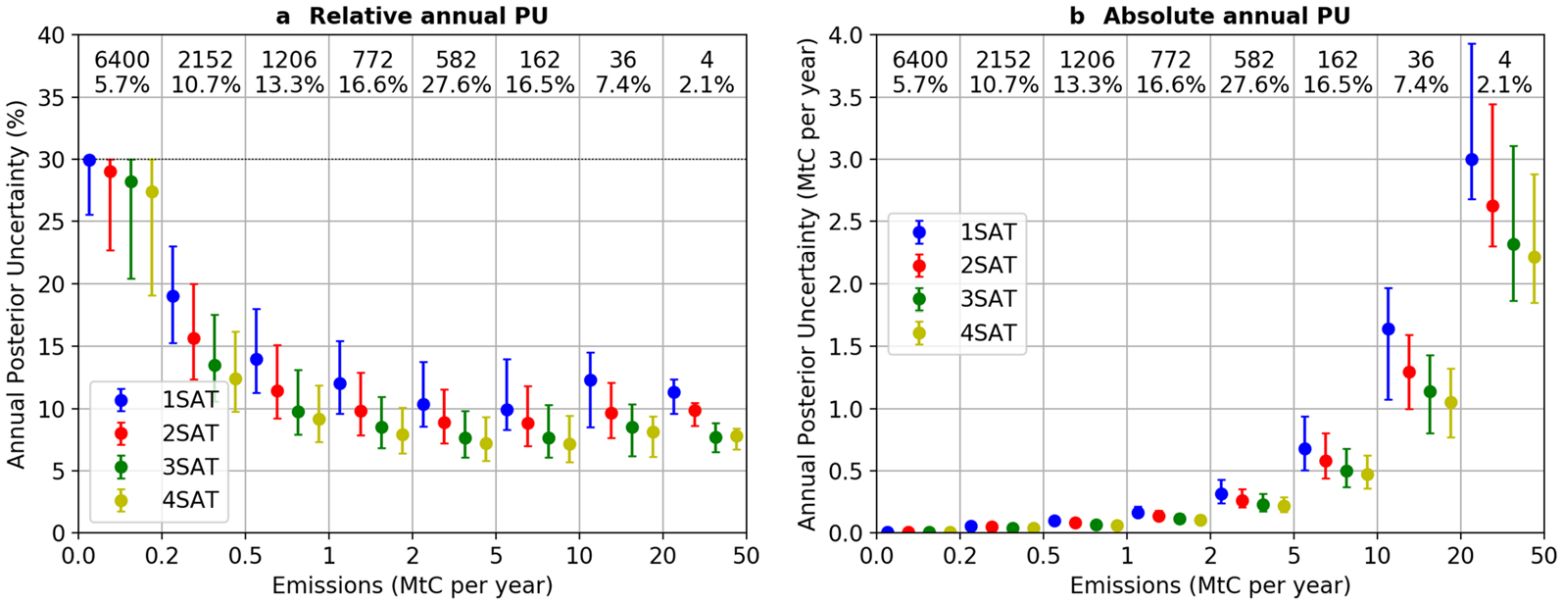
Same as Fig. 2 but for relative (**a**) and absolute (**b**) posterior uncertainty in annual CO_2_ emissions for clumps in INV-annual

Figure 4 shows the median and interquartile range of the annual posterior uncertainty. It shows that the annual posterior uncertainty values are less than 20% only for clumps whose annual emission budget is larger than 0.2 MtC per year. These clumps account respectively for 43.4% and 94.3% of the total number of clumps and of the fraction of total CO_2_ emissions covered by all clumps. The annual posterior uncertainty tends to decrease with increasing clump emissions, highlighting, as expected, that emissions from large clumps are easier to constrain. Similar patterns are observed for all regions of the globe (Additional file 1: Fig. S3) and agree with the results found by Wang et al. [35] with one satellite. Increasing the number of satellites beyond one satellite allows to further reduce the annual uncertainty on CO_2_ emissions for all clumps, but the gain obtained on the annual posterior uncertainty is within a few percent between *n* and *n* + *1* satellites, with *n* = 1, 2, 3. For example, one satellite can constrain the uncertainty in annual budget from 30% (prior) to 12% (posterior) for the 1–2 MtC per year emission bin (Fig. 4), while 2, 3 and 4 satellites constrain the annual posterior uncertainty to 9.8%, 8.5% and 7.9%.

**Fig. 5:**
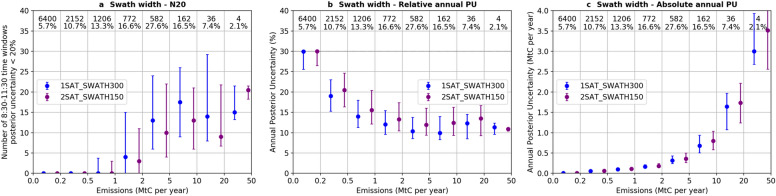
**a** Number of 8:30–11:30 time windows in a year for which the posterior uncertainty in the 3 h mean emissions are less than 20% (N20) in INV-3 h; **b** Relative posterior uncertainty in annual CO_2_ emissions in INV-annual for 1 satellite with a swath width of 300 km (blue) and for 2 satellites with a swath width of 150 km (purple); and **c** the same as **b** but for absolute values of posterior uncertainty. The results are binned according to the clump annual emission with bin limits given on the x-axis of the figure. Numbers within the figure indicate the number of clumps in the bin and the fraction of total CO_2_ emissions generated by the clumps. Dots and error bars are the median and interquartile range of N20 (**a**) and posterior uncertainty (**b**)

Figure 5 shows the median and interquartile range of N20 (Fig. 5a) and annual posterior uncertainty (Fig. 5b and 5c) for the 300-km swath width 1-satellite constellation (1SAT_SWATH300) and for the 150-km swath width 2-satellite constellation (2SAT_SWATH150). The results show that multiplying the number of satellites while reducing the swath width by a factor of two has a negative impact on the performance of inversions for most bins of clumps. For example, the N20 median values are respectively 4/3, 13/10, 18/13, 14/9 for the 1–2, 2–5, 5–10 and 10–20 MtC per year emission bins for 1SAT_SWATH300/2SAT_SWATH150 (Fig. 5a). As a consequence, the annual posterior uncertainty are lower for 1SAT_SWATH300 than for 2SAT_SWATH150. Having larger images with the 300 km swath allows to catch much larger portions of the plumes during the overpass which appears to be critical to pass the threshold of the 20% posterior uncertainties for most of the emission bins. The differences between the two constellation configurations are however less than a few percent (Fig. 5b) within each emission bin. There is an exception for the strongest emission bin (20–50 MtC per year) for which the N20 (annual posterior uncertainty) values are higher (lower) for 2SAT_SWATH150 than for 1SAT_SWATH300, probably due to the statistical artefact of having only four clumps in this bin.

**Fig. 6:**
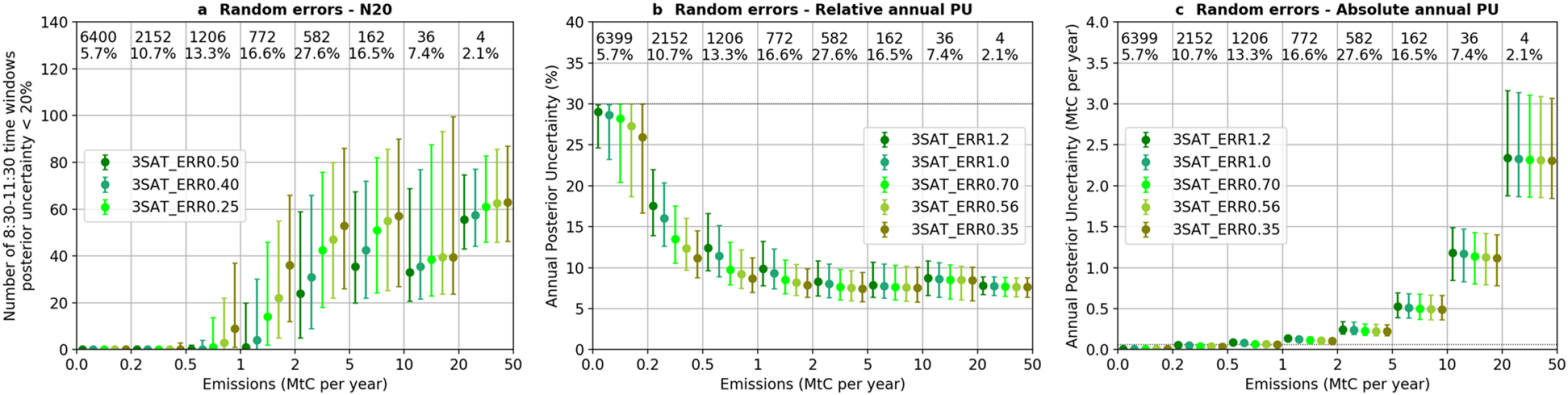
Same as Fig. 5 but for different scenarios of random errors

Figure 6 shows the median and interquartile range of N20 (Fig. 6a) and annual posterior uncertainty (Fig. 6b and 6c) with different random measurement error for the three-satellite constellation. The results show that, as expected, increasing the precision of the instruments leads to an increase of the N20 values for most clumps. The increase is, however, more significant for the clumps whose annual emission budget is between 0.5 – 5 MtC per year than for those with larger annual emission budget. When the random measurement error is 1.2 ppm for vegetation albedo and SZA 50º, the N20 values for the sources within 0.5–1 MtC per year emission bin decreases to zero. In addition, the annual posterior uncertainty decreases when random error is smaller, but the differences between the random error scenarios are of the order of a few tenths of a percent within each emission bin.

## Discussion

PMIF was designed to permit a global evaluation of the impact of different configurations of satellite constellations to constrain the CO_2_ emissions from localized sources. Although some sources of uncertainties were not accounted for, such as diffuse emissions, the effect of natural CO_2_ fluxes, systematic measurement error, and error in using Gaussian plume model to represent atmospheric transport, PMIF can be used to investigate the first-order impacts of the configurations of satellite instruments and other key parameters in the inversion system, such as temporal error autocorrelation of the uncertainties in the emissions of clumps.

Our results confirm that the potential of a constellation to monitor CO_2_ emissions is highly dependent on the level of emissions of the clumps, as was shown by Wang et al. [35] with one satellite. It is found for all configurations with different numbers of satellites and instrument precision, a small city or thermal power plant with an annual budget lower than 0.5 MtC per year will not have any chance to have its emissions during 8:30–11:30 time windows (in this study, e.g. 3 h before satellite overpasses) constrained to better than 20% by the satellite imagers like those planned for CO2M. Although the small clumps that will remain below the detection threshold are numerous (75.6% of all clumps), they only represent 16.4% of the total clump CO_2_ emissions. Conversely, clumps whose emissions are higher than 0.5 MtC per year have at least one 3 h (8:30–11:30) time window during which the emissions can be significantly constrained with a posterior uncertainty less than 20%. The N20 values almost linearly increase with the number of satellites for all bins of clumps > 0.5 MtC per year. Improving random measurement error can also lead to an increase in the N20 values for clumps with emissions between 0.5–5 MtC per year, but its impact is relatively small compared to that of the number of satellites. Physically, the increase in the number of satellites increases the revisit frequency, and thus increase the N20 values, while an increase in the random measurement error will only improve the constraints on the same days as the reference inversion, resulting more precise posterior estimate (i.e. with smaller posterior uncertainty) of the 3 h emissions.

Because more satellites provide constraints on the 3 h emissions on more days, it is expected that the posterior uncertainty of the annual emission will improve (i.e. decreases) as the number of satellites increases. At the same time, because more precise observations lead to more precise estimates of the 3 h emissions, it is also expected that these improved estimates of 3 h emissions could provide improved information about the clump emissions at longer period (e.g. annual emissions). Our results confirm this expected results, but both the increase in the number of satellites and the improvement in the precision of individual XCO_2_ retrievals only have marginal benefit. This is due to the fact that the CO2M satellite observations only provide direct constraints on the emissions during 3 h before the overpasses for a limited number of days. The emissions is not directly constrained during the remaining 21 h and during the 8:30–11:30 on other days with no satellite observation sampling the plumes of clump emissions. In general, there are less than 70 occurrences when the emissions during 8:30–11:30 are constrained (Fig. 6a, dark green dots), the emissions in these time windows sum up to less than 10% of the annual emissions. The inversion system extrapolates the information on the emissions in 3 h time windows obtained from the observations, through the temporal auto-correlations between the prior uncertainties in emissions, to constrain the remaining 90% emissions. But the reliability of such extrapolation stays rather weak, leaving large residual error. This residual error, especially that in the emissions in the afternoon and night, will not reduce by simply adding more satellites sampling the plumes of emissions generated by emissions in the morning time (i.e. 8:30–11:30) or by improving the measurement precision. A combination of various LEO constellations with different crossing time could provide more information on the emissions during different times of the day, and thus further improve the inversion performance. The geostationary-orbit (GEO) imagers, such as GeoCARB [19, 23] or other GEO concepts [27, 28], can also offer frequent sampling of the plumes to constrain the diurnal variations in the emissions. Satellites in a highly elliptical orbit (HEO) could provide continuous or quasi-geostationary coverage of high latitudes [21]. GEO and HEO satellites are thus also part of the long-term vision of Europe [26] and of the Committee on Earth Observation Satellites (CEOS) Constellation Architecture [9] for CO_2_ monitoring from space. On the other hand, Wang et al. [35] showed that the extrapolation from 3 h emissions to annual emissions is highly dependent on the assumption of temporal correlation between prior uncertainties. However, the temporal auto-correlations are poorly known in the emission inventories, and previous studies selected the temporal auto-correlations in the prior uncertainty arbitrarily [16, 17]. Wang et al. [35] also showed that the exponential function commonly used by the inversion community might be a poor representation of such temporal auto-correlations, highlighting the need of systematic assessments of the uncertainties in the emission products and its error structures.

Lastly, our study shows that having larger swath for individual satellites is advantageous to having more satellites for the monitoring of clump emissions at both 3 h and annual scales (Fig. 5). Having larger swath and having more satellites will both increase the sampling frequency, but the former option also allows for sampling a large portion of the plumes generated by clumps. Our study focuses on the use of data systematically taken on nadir mode, which the most favorable over land [18]. The monitoring of sources located near coasts could benefit from observations in glint mode. However, the width of the glint spot of CO2M should hardly exceed 30 km, which hampers the characterization of the plumes. Furthermore, this limitation of the effective images indicates that the benefit of having larger swath would be lost for the glint mode, for which having more satellites would be more critical. In addition, the observations in glint mode will also help in the global large-scale inversions of natural fluxes, especially over the ocean, in which case it is more useful to have more satellites. It thus suggests that in the design of satellite missions, one should balance the costs among having more satellites, larger swath, and smaller measurement error, taking into account for the main target of the missions.

This study has investigated the capability of satellite imagers to quantify the fossil fuel CO_2_ emissions from large cities and point sources over one year. The capability of these satellites to quantify long-term trends of emissions over several years has not been investigated. Wang et al. [33] showed that the uncertainties in the trends of emissions are proportional to the uncertainties in the emissions of individual years. Qualitatively, the potential to estimate the emissions in the morning time increases proportionally increase with the number of satellites (Fig. 2), implying that the long-term trends in emissions in the morning time can be better estimated with more satellites. However, Fig. 4 showed that the posterior uncertainties in annual emissions are close to each other when having one to four satellites, indicating that the gain of having more satellites to estimate the trends in annual emissions are limited. This is related to the fact that there are large hour-to-hour, day-to-day, month-to-month and year-to-year variations in the emissions. The limited number of 3 h time windows for which the inversion yields small posterior uncertainties may hardly help distinguish these different sources of variations for the window 8:30–11:30, and even less for the emissions in the night and afternoon. To properly estimate the potential to estimate emission trends as a function of the number of satellites, multi-year inversions accounting for these different sources of variations would be needed.

## Conclusions

In this study, we use the PMIF global inversion system to assess the performance of a constellation of one to four CO2M imagers to monitor anthropogenic CO_2_ emissions. Given the typical measurement precision of individual retrievals of XCO_2_, the plumes from emission sources with an annual emission smaller than 0.5 MtC can hardly be detected by satellite imagers. The number of time windows during which the emissions can be significantly constrained with a posterior uncertainty less than 20% is proportional to the number of satellites for clumps with an annual emission larger than 0.5 MtC.

The XCO_2_ observations from satellite imagers could provide direct constraints on the estimate of emissions before the overpasses on clear-sky days, representing less than 10% of the annual emissions for a single clump. The emissions during other times are constrained through the temporal auto-correlations between the prior uncertainties. Improving the precision of individual retrievals will significantly improve the potential to constrain the emissions that are already well constrained in the reference simulation, whereas the improvement on the extrapolation of the constraints on the estimate of emissions in the afternoon and night is limited. As a result, the potential of the satellite imagers to monitor annual emissions is not proportional to the precision of individual retrievals. This study aslo shows that having larger swath for individual satellites is advantageous to having more satellites with narrower swaths.

## Supplementary information

**Additional file 1.** Additional figures and tables.

## Data Availability

The source code for PMIFv1.0 for inversions with one satellite is published in Wang et al. [35]. The ODIAC inventory is available at https://db.cger.nies.go.jp/dataset/ODIAC/DL_odiac2018.html. The clump dataset is available at https://doi.org/10.6084/m9.figshare.7217726.v1. The wind fields from CCMP are available at https://www.remss.com/measurements/ccmp/. EDGAR v4.3.2 emission maps are needed to run the SectCS inversion, and are available at https://edgar.jrc.ec.europa.eu/overview.php?v=432_GHG.

## Abbreviations

LEO: Low-Earth orbit
ESA: European Spatial Agency
CO2M: Carbon dioxide monitoring
XCO_2_: Vertically integrated columns of dry air mole fractions of CO_2_
PMIF: Plume-monitoring inversion framework
ODIAC: Open Source Data Inventory of Anthropogenic CO_2_
CCMP: Cross-calibrated multi-platform
AMS: Annual component and moderately correlated Sub-annual component
SZA: Solar zenith angle
MODIS: Moderate Resolution Imaging Spectroradiometer
OSSE: Observing system simulation experiment
