# Supplementary material for: The potential of a constellation of low earth orbit satellite imagers to monitor worldwide fossil fuel CO2 emissions from large cities and point sources
Source: Carbon Balance Manag. 2020 Sep 4;15:18. doi: 10.1186/s13021-020-00153-4 (PMC7650226; doi:10.1186/s13021-020-00153-4)
Supplement: Supplementary file 1 — Additional file 1. Additional figures and tables. [file 13021_2020_153_MOESM1_ESM.docx]

Additional Material for

The potential of a constellation of low earth orbit satellite imagers to monitor worldwide fossil fuel CO_2_ emissions from large cities and point sources

Franck Lespinas^1,2^, Yilong Wang^1,3*^, Grégoire Broquet^1^, François-Marie Bréon^1^, Michael Buchwitz^4^, Maximilian Reuter^4^, Yasjka Meijer^5^, Armin Loescher^5^, Greet JanssensMaenhout^6^, Bo Zheng^1^, Philippe Ciais^1^

^1^Laboratoire des Sciences du Climat et de l’Environnement, CEA-CNRS-UVSQ - Université Paris Saclay, 91191, Gif-sur-Yvette CEDEX, France

^2^Canadian Centre for Meteorological and Environmental Prediction, 2121 Transcanada Highway, Dorval, QC, H9P 1J3, Canada

^3^The Key Laboratory of Land Surface Pattern and Simulation, Institute of Geographical Sciences and Natural Resources Research, Chinese Academy of Sciences, Beijing, China

^4^Institute of Environmental Physics (IUP), University of Bremen FB1, Otto Hahn Allee 1, 28334 Bremen, Germany

^5^European Space Agency (ESA), Noordwijk, Netherlands

^6^European Commission, Joint Research Centre, Directorate Energy, Transport & Climate, Via Fermi 2749, 21027 Ispra, Italy

^*^*Correspondence to*: Yilong Wang (wangyil@igsnrr.ac.cn)

**Contents of this file**

Supplementary Figures S1-S3


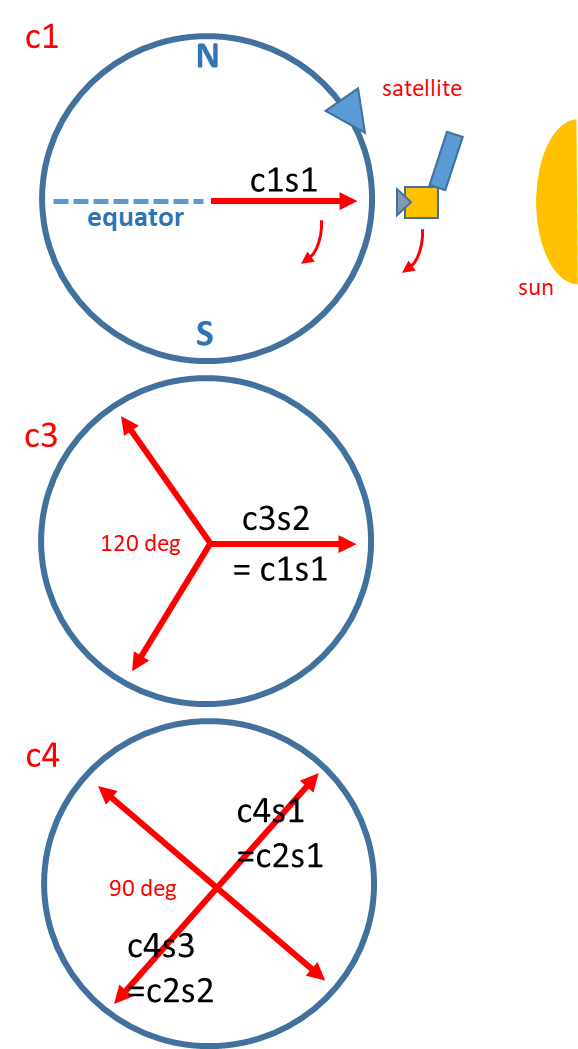


**Figure S1** Regular positioning of the satellites of the CO2M constellations on the chosen helio-synchronous orbit. The notations “c1”, “c3” and “c4” denote the number of satellites in the constellation, and “s1” and “s2” denote the index of satellites in each constellation. Note that the 2^nd^ satellite (c3s2) in c3 is identical to the satellite in c1, and the 1^st^ and 3^rd^ satellites in c4 are identical to the two satellites in c2.


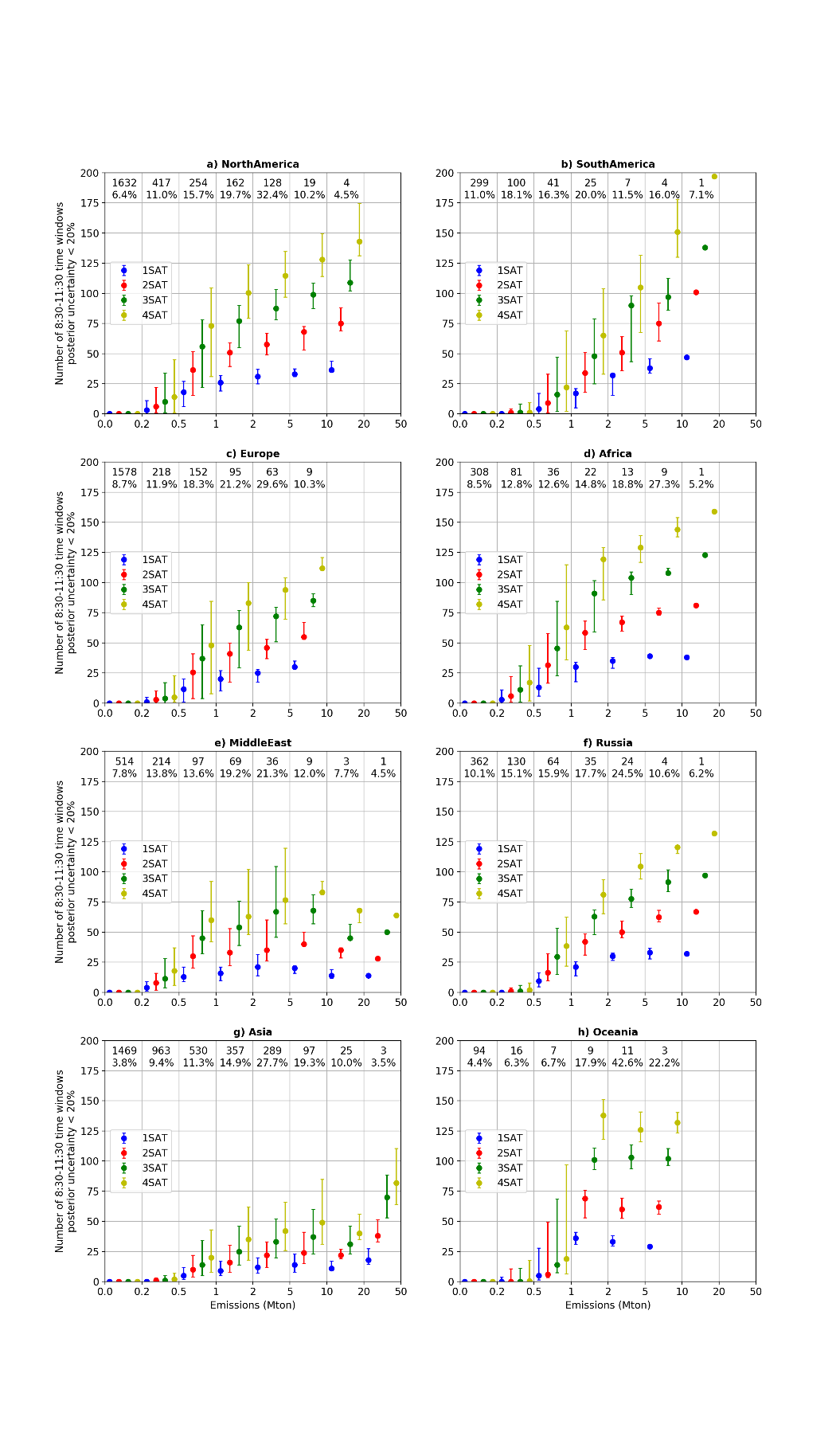


**Figure S2** Number of 8:30-11:30 time windows in a year (here for the year 2008) for which the posterior uncertainty in the 3 h mean emissions are less than 20% (N20) for different regions of the globe in INV-3h. The results are binned according to the clump annual emission with bin limits given on the x-axis of the figure. Numbers within the figure indicate the number of clumps in the bin and the fraction of total CO_2_ emissions from all the clumps that they represent. Dots and error bars are the median and interquartile range of N20.


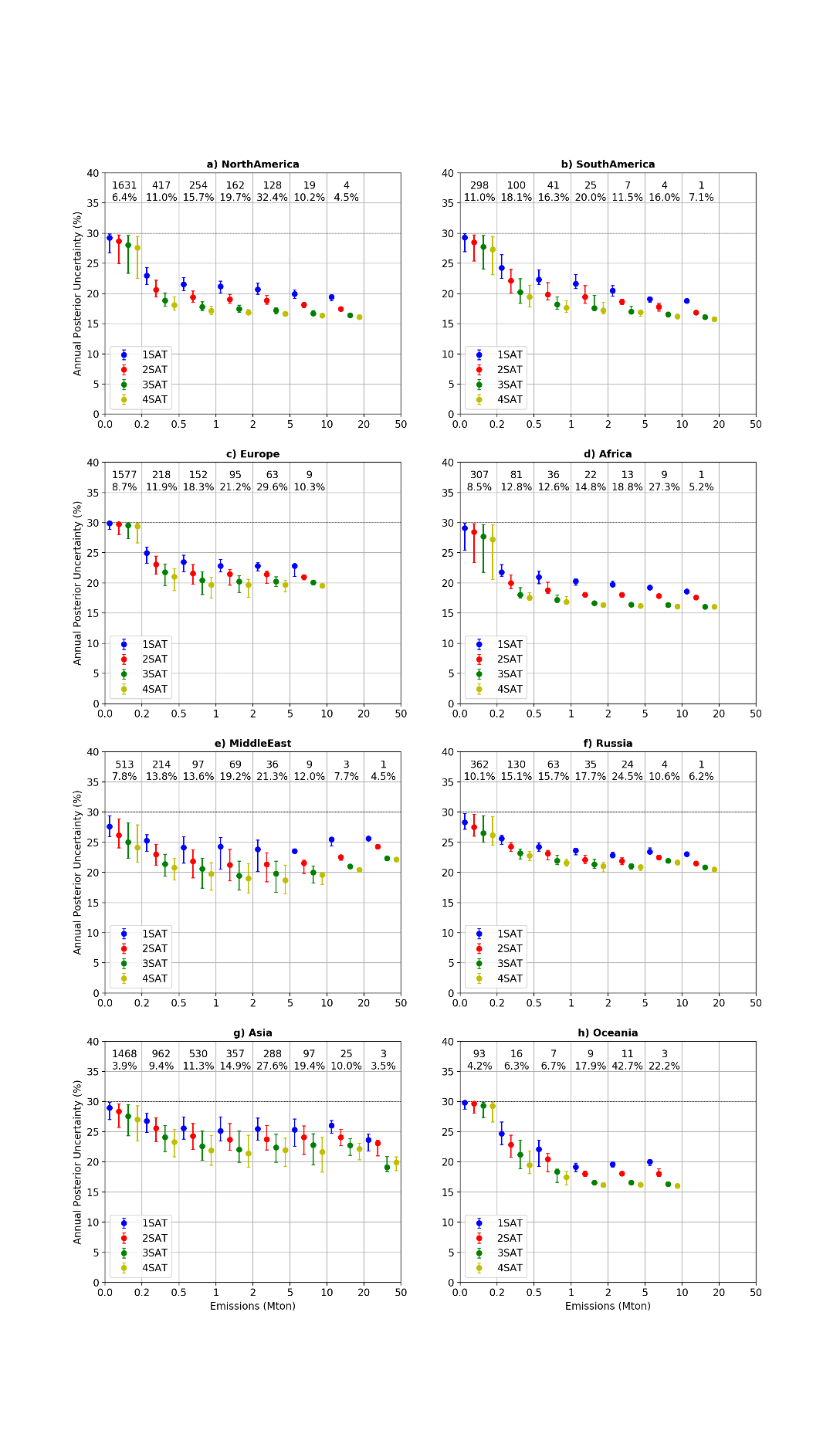


**Figure S3** Posterior uncertainty in annual CO_2_ emissions for clumps in different regions of the globe in INV-annual. The results are binned according to the clump annual emission with bin limits given on the x-axis of the figure. Numbers within the figure indicate the number of clumps in the bin and the fraction of total CO_2_ emissions generated by the clumps. Dots and error bars are the median and interquartile range of PU.
